# FISH Diagnostic Assessment of *MDM2* Amplification in Liposarcoma: Potential Pitfalls and Troubleshooting Recommendations

**DOI:** 10.3390/ijms24021342

**Published:** 2023-01-10

**Authors:** Alessandro Gambella, Luca Bertero, Milena Rondón-Lagos, Ludovica Verdun Di Cantogno, Nelson Rangel, Chiara Pitino, Alessia Andrea Ricci, Luca Mangherini, Isabella Castellano, Paola Cassoni

**Affiliations:** 1 Division of Liver and Transplant Pathology, University of Pittsburgh, Pittsburgh, PA 15213, USA; 2 Department of Medical Sciences, University of Turin, 10124 Turin, Italy; 3 School of Biological Sciences, Universidad Pedagógica y Tecnológica de Colombia, Tunja 150003, Colombia; 4 Department of Laboratory Medicine, Azienda Ospedaliera Città della Salute e della Scienza di Torino, 10126 Turin, Italy; 5 Departamento de Nutrición y Bioquímica, Facultad de Ciencias, Pontificia Universidad Javeriana, Bogotá 110231, Colombia

**Keywords:** *MDM2* amplification, liposarcoma, FISH, *MDM2* interpretation guidelines

## Abstract

*MDM2* amplification represents the leading oncogenic pathway and diagnostic hallmark of liposarcoma, whose assessment is based on Fluorescence In Situ Hybridization (FISH) analysis. Despite its diagnostic relevance, no univocal interpretation criteria regarding FISH assessments of *MDM2* amplification have been established so far, leading to several different approaches and potential diagnostic misinterpretations. This study aims to address the most common issues and proposes troubleshooting guidelines for *MDM2* amplification assessments by FISH. We retrospectively retrieved 51 liposarcomas, 25 Lipomas, 5 Spindle Cell Lipoma/Pleomorphic Lipomas, and 2 Atypical Spindle Cell Lipomatous Tumors and the corresponding *MDM2* FISH analysis. We observed *MDM2* amplification in liposarcomas cases only (43 out of 51 cases) and identified three *MDM2*-amplified patterns (scattered (50% of cases), clustered (14% of cases), and mixed (36% of cases)) and two nonamplified patterns (low number of signals (82% of cases) and polysomic (18% of cases)). Based on these data and published evidence in the literature, we propose a set of criteria to guide *MDM2* amplification analysis in liposarcoma. Kindled by the compelling importance of *MDM2* assessments to improve diagnostic and therapeutic liposarcoma management, these suggestions could represent the first step to develop a univocal interpretation model and consensus guidelines.

## 1. Introduction

Within the neoplastic soft tissue panorama, liposarcoma (LPS) represents the most common type of adult sarcoma, accounting for almost 20% of cases worldwide [1,2]. LPS is a clinical challenge, as it presents a high recurrence rate, unsatisfactory response to available treatments, and a challenging diagnostic workup, especially if based on morphology and immunohistochemistry alone [3].

The new WHO Classification of Soft Tissue Tumors edition identifies several specific subtypes of LPS, namely Atypical Lipomatous Tumors (ALT)/Well-Differentiated LPS (WDLPS), Myxoid LPS (MLPS), Dedifferentiated LPS (DDLPS), and Pleomorphic LPS (PLPS) [4,5,6]. The most common variants are WDLPS and MLPS, while DDLPS represents the aggressive evolution of WDLPS [7]. From a pathologist perspective, WDLPS and DDLPS represent the most challenging variants, as WDLPS can present overlapping features with benign tumors such as Lipomas, while DDLPS may show extensive dedifferentiated areas that are morphologically indistinguishable from other high-grade sarcomas [8,9,10]. Additionally, these neoplasms are usually initially approached with a needle biopsy, further complicating pathologist’s evaluation because of the limited sample available. Although immunohistochemistry (IHC) represents a valuable diagnostic tool for multiple sarcoma types, unfortunately, no immunophenotype specifically defines LPS, limiting its usefulness in this setting [8,11].

**Figure 1:**
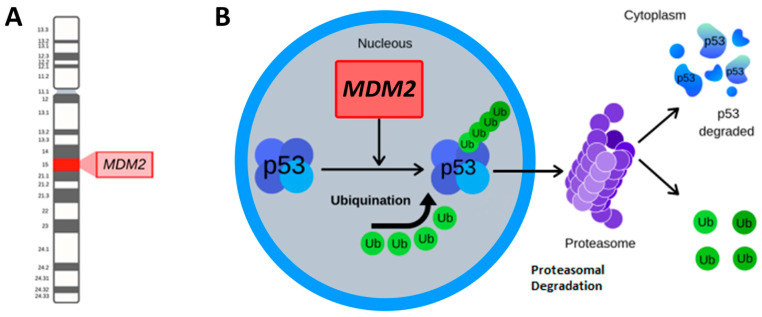
*MDM2* location and function. (**A**) *MDM2* is located in the long arm of chromosome 12, region 1, and band 5 (12q15). (**B**) *MDM2* is an E3 ubiquitin ligase that targets p53 for ubiquitylation and subsequent proteasomal degradation.

Molecular testing is a reliable and informative analysis to achieve an LPS diagnosis. The amplification of the *Murine Double Minute-2* (*MDM2*) gene emerged as an essential molecular hallmark of LPS, particularly for ALT/WDLPS and DDLPS. *MDM2* is an oncogene located in the long arm of chromosome 12 (12q15) (Figure 1) and its activity is strictly related to the expression of p53, with a negative feedback-type relationship.

MDM2 acts as an E3 ubiquitin-ligase, binding p53 and promoting its ubiquitylation and consequent proteasome-dependent degradation [12] (Figure 1). Low levels of *p53* activity are unable to (I) regulate the cell cycle and (II) induce apoptosis in DNA-damaged cells, leading to uncontrolled proliferation. On the other hand, *p53* regulates the expression of *MDM2*, balancing its activity and influence on the cell cycle and proliferation. MDM2 also regulates the Retinoblastoma protein (RB) [13]. In human tumor cell lines, MDM2 enhances RB degradation through a proteasome-dependent mechanism in a similar process as seen for p53 [12].

*MDM2* amplification emerged as an oncogenic pathway in several malignances, but it is mostly represented in soft tissue sarcomas [14,15,16,17] where, differently from other tumors, *MDM2* amplification and p53 mutation are mutually exclusive [18,19,20,21]. In soft tissue sarcomas, the *MDM2* amplification occurs mainly through the so-called double minutes chromosomes (Dmins) mechanism [22]. Dmins are small, generally acentric, and autonomously replicating chromatin bodies that act as an amplification mechanism for several oncogenes, including *MDM2* [23,24,25,26]. The sarcomas showing the highest percentages of *MDM2* amplification are the low-grade/periosteal osteosarcoma, ALT/WDLPS, DDLPS, and intimal sarcoma [27]. Indeed, the evaluation of the *MDM2* status is fundamental in the LPS diagnostics workup, as its amplification is present in 95% of WDLPS and DDLPS cases, while benign lipomatous lesions show no amplification at all [9,10,12]. IHC for *MDM2* expression is available and frequently (>95% of cases) positive in WDLS. However, previous reports found an unsatisfactory correlation between IHC for the MDM2 protein and *MDM2* gene amplification status, particularly in poorly differentiated cases or in cases with *MDM2* overexpression not related to gene amplification [8,11,27,28,29]. Considering the clinical consequences of misdiagnosing these lesions, the molecular analysis of *MDM2* is essential, and it is usually performed with Fluorescence In Situ Hybridization (FISH) [30]. Indeed, the *MDM2* amplification evaluation by FISH is a crucial and well-established assay for liposarcoma diagnostic work-up and, nowadays, is considered as a diagnostic gold standard [31,32,33]. However, despite its diagnostic relevance, no definitive consensus has ever been defined for determining the *MDM2* gene status. Considering the frequency of LPS in the adult population and the diagnostic and prognostic implications, the establishment of interpretation guidelines is an unmet need of crucial importance.

In this setting, our study aims to improve the diagnostic interpretation of the FISH assessment of MDM2 amplification in LPS by evaluating the potential drawbacks and pitfalls and suggesting a potential set of diagnostic criteria to achieve a standardized evaluation of this diagnostic test.

## 2. Results

### 2.1. MDM2 Amplification Accurately Stratify Our Series

We collected and analyzed 27 DDLPS, 19 WDLPS/ALT, 3 PLPS, 2 MLPS, 25 Lipomas, 5 Spindle Cell Lipoma/Pleomorphic Lipomas (SLC/PL), and 2 Atypical Spindle Cell Lipomatous Tumors (ASCLT). In our series, 43 cases (52%) presented *MDM2* amplification including 25/27 DDLPS (93%) and 18/19 WDLPS/ALT (95%).

Amplified DDLPS cases presented a mean *MDM2/CEP12* ratio of 10.1 (range 4.0–20.3) further detailed in a mean *MDM2* copy number per cell of 24.1 (range 13.9–30.2) and a mean *CEP12* copy number per cell of 2.5 (range 1.4–3.5).

WDLPS amplified cases presented a mean *MDM2/CEP12* ratio of 8.2 (range 4.8–16.0). The mean *MDM2* copy number per cell was 17.9 (range 11.4–28.4), and the mean *CEP12* copy number per cell was 2.4 (range 1.8–3.7). The results are detailed in Table 1.

### 2.2. MDM2 Amplification Patterns

**Figure 2:**
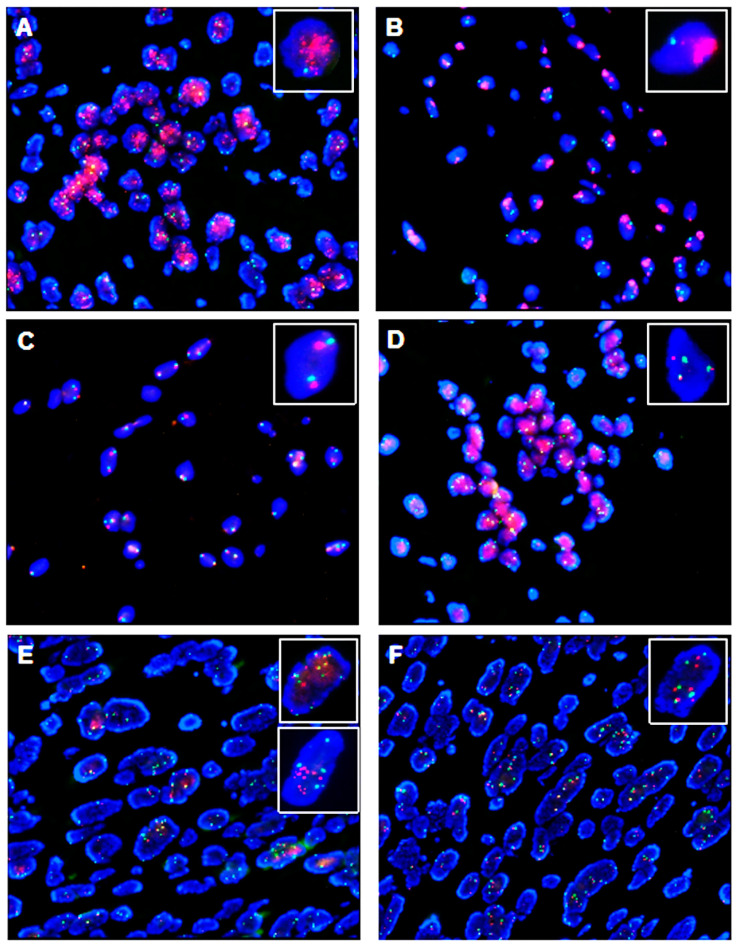
Patterns of *MDM2* gene status by FISH assessment. MDM2 red signals and CEP12 green signals (Abbott molecular probes). Nuclei are counterstained with 4′-6-diamino-2-phenylindole and appear blue. (**A**) MDM2 amplification with MDM2 > 10 and CEP12 = 2. Notably, several MDM2 gene dot-like signals are scattered over the whole nucleus. Focal areas with overlapping signals are also present. Overall, this pattern is consistent with gene localization on Dmin. (**B**) MDM2 amplification with several areas with crowded, overlapping signals arranged in clusters over the whole nucleus and two CEP12 signals. This pattern is typical of Dmin amplification as well. (**C**) No MDM2 amplification nor CEP12 augmented copies are present in interphase of tumor cells’ nuclei (negative case). (**D**) An increased number of both MDM2 and CEP12 signals (3–4 copies) in a polysomic sample which resulted negative for MDM2 amplification. (**E**) A challenging sample with the presence of “giant nuclei”: a polysomic non-MDM2-amplified nucleus with more than ten MDM2 and CEP12 signals is shown in the top inset; for comparison, the lower inset presents an *MDM2* amplified nucleus from another case with MDM2 ≥ 10 and a ratio > 2. (**F**) EGFR (red signals) and CEP7 (green signals) probes in “giant nuclei” (same case of 3E).

*MDM2* amplified cases presented a high number of *MDM2* copies (mean 21; range 11–30.2), a low number of *CEP12* (mean 2.5; range 1.4–3.7), and, consequently, an *MDM2/CEP12* ratio > 2. *MDM2* positive cases presented three distinctive FISH amplification patterns: (i) the first (50% of cases) was characterized by several scattered signals distributed over the whole nucleus (Figure 2A); (ii) the second pattern (14% of cases) presented *MDM2* signals clustered in specific areas of the nucleus (Figure 2B); (iii) the third presented overlapping features of the two previous patterns, as signals were contemporarily clustered and scattered (36% of cases). Regardless of these patterns, *MDM2* amplified cases also presented extra chromosomal signals that were referred to as being Dmin. These signals were so small that they could be read by the Metafer 4 in 18 of the 43 amplified cases (42%) only. Of note, the Dmin amplification could present focal areas with overlapping signals resulting in an overall blurry appearance [34,35,36,37].

On the other hand, cases resulting negative for *MDM2* amplification presented two different patterns: (i) The most common one (82% of cases) showed less than three *MDM2* gene signals (*MDM2* < 3; mean 2.2; range 1.7–2.9). These cases were considered negative regardless of the *MDM2/CEP12* ratio (Figure 2C). (ii) The second pattern (18% of cases) showed instead three or more *MDM2* gene signals (*MDM2* ≥ 3; mean 3.7; range 3.1–4.3) together with a gain of the chromosome 12 centromere (mean 3.1; range 2.0–3.9) (Figure 2D).

We also identified two subgroups of cells with “giant nuclei” (i.e., at least two times larger than nearby nuclei) that presented challenging features. The first subgroup consisted of chromosome 12 polysomy (more than ten *MDM2* and *CEP12* signals) (Figure 2E—top inset) that was negative for *MDM2* amplification by ratio. The evaluation of the *EGFR/CEP7* dual probe expression in this subgroup revealed *EGFR/CEP7* polysomy and confirmed the cells’ polyploidy (Figure 2F). The second subgroup is represented by amplified cells with “giant nuclei” harboring increased *MDM2* gene signals and was positive by ratio (Figure 2E—bottom inset).

### 2.3. Literature Review and Analysis

We first identified 90 studies that initially satisfied the keywords research on main databases. After a careful analysis and screening, 36 studies were eventually selected (Table 2).

**Figure 3:**
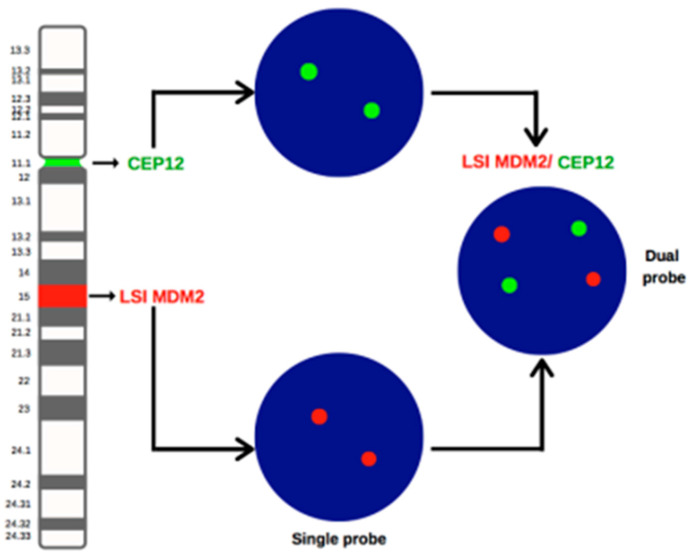
Schematic representation of the probes used for *MDM2* gene evaluation and the expected FISH pattern. CEP12: centromeric probe for chromosome 12; LSI MDM2: Locus Specific Identifier for MDM2 gene; LSI MDM2/CEP12: dual probe.

From these studies, a relevant heterogeneity in the *MDM2* amplification assessment clearly emerged, as the median number of evaluated nuclei per analysis was 60 but with a wide range (20 to 200). Similarly, the definition of *MDM2* amplification itself varied significantly, as some studies based their evaluation upon the absolute *MDM2* count (i.e., three or more signals as sufficient for a diagnosis of amplification [51,63], while others set the limit to five signals) [30,52,54,57]. More commonly, the *MDM2/CEP12* ratio was used, but again with variable cut-off thresholds including >2 [10,41,44,46,47,50,59], ≥2 [8,9,42,43,48,49,56,61], or ≥2.2 [58], probably borrowing this criteria from the previous ASCO/CAP interpretation guidelines for *HER2* assessment [64]. Of note, even the type of FISH probes utilized greatly differed or was not exhaustively reported. FISH probes ranged from home made [8,9,10,48] to commercially available, the latter including dual probes (LSI *MDM2/CEP12*) [30,44,46,47,49,50,51,52,56,57,58,59,61] but also single locus probes (LSI *MDM2*) that do not allow chromosome 12 polysomy evaluation [1,54,56] (Figure 3).

## 3. Discussion

To date, soft tissue lesions are initially approached and diagnosed mainly through the accurate combination of clinical data and morphological features of biopsy samples. This practice, although useful and accurate in several settings, presents crucial drawbacks, especially when dealing with equivocal and misleading histopathological findings, as in the LPS scenario. Indeed, LPS and, particularly, the ALT/WDLPS, DDLPS, and UPS variants present relevant overlapping morphologic features with other types of malignant sarcomas or even with benign entities, such as Lipomas, thus leading to a challenging diagnostic assessment and a broad differential diagnosis process. In this context, the evaluation of *MDM2* status is of crucial support, as it represents a diagnostic hallmark of LPS. Regardless of its diagnostic relevance, no guidelines or consensus criteria regarding *MDM2* gene amplification interpretation have ever been proposed, whereas previous studies assessing this molecular hallmark adopted significant methodological differences, including a broad spectrum of criteria for probe counting and diagnostic cut-off values [65,66]. This methodological heterogeneity could lead to confounding definitions and hamper the diagnostic reliability/reproducibility, eventually misleading patient clinical management.

**Figure 4:**
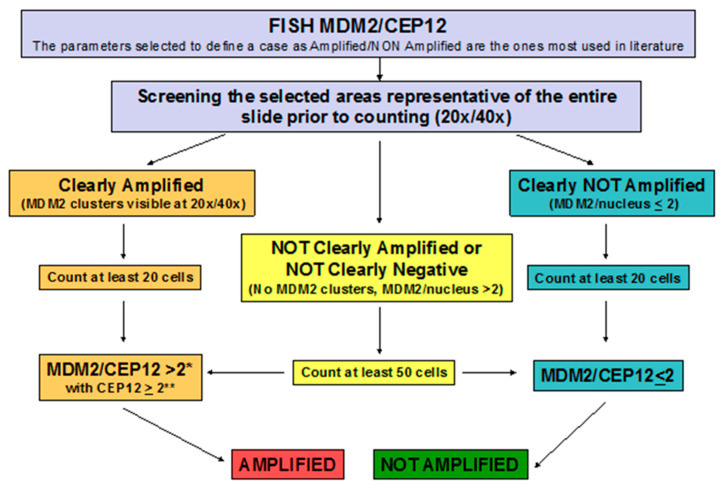
Criteria settled for FISH MDM2/CEP12 analysis. * Generally, cases with MDM2 amplification have a lot of MDM2 signals, so we think that an MDM2/CEP12 ratio > 2 is better than MDM2/CEP12 ≥ 2 as a diagnostic criterion. ** This parameter supports excluding cases with loss of CEP12 (CEP12 per nucleus < 2) as amplified.

Based on published evidence and our data, we hereby provide a set of considerations and recommendations regarding FISH interpretation criteria for *MDM2* amplification assessments that should be considered in the LPS diagnostic workup (Figure 4).

#1 The analyzed nuclei must be representative of the entire lesion. The FISH analysis is performed in a small, selected area, that, however, has to be representative of the whole lesion. We recommend that an expert pathologist in soft tissue tumors determines the area for FISH analysis, supplying marked hematoxylin–eosin (H&E)-stained slides. This criterion, common to every molecular analysis, should be strictly and routinely applied to avoid misleading results due to nonrepresentative sampling.

#2 The *MDM2* amplification is secondary to a Dmin-based mechanism. Dmins are extra chromosomal elements that are considered cytogenetic hallmarks of high-gene amplification. Based on our data, Dmins were the main mechanism behind *MDM2* amplification in all the cases evaluated.

#3 *MDM2/CEP12* ratio is essential to define *MDM2* status. We recommend determining the *MDM2/CEP12* ratio and consider only cases with a ratio > 2 as amplified. Generally, *MDM2* amplified cases present many *MDM2* gene signals together with a mean of two CEP12 signals. In our experience, observing a >2 *MDM2/CEP12* ratio is necessary to differentiate amplified from polysomic cases. We did not experience cases with a ratio = 2, but we consider that this equivocal pattern could be solved by extending the cells count. Furthermore, we observed no cases with *MDM2* signals < 4 and a ratio > 2. This eventuality could happen due to the loss of the *CEP12* (*CEP12* < 1.8), but we would have considered these cases as not amplified without any further analysis. Similarly, cases with several *MDM2* signals (e.g., >6) but a <2 ratio should be considered nonamplified as well. In this scenario, the use of other enumeration probes could prove helpful in confirming cells’ polysomic nature.

#4 Ambiguous cases require attention and critical review, especially if polysomy is suspected. In ambiguous cases and, in particular, if polysomy is suspected, we recommend analyzing at least 50 nuclei to avoid *MDM2* status misinterpretation. In these cases, a careful assessment of the *MDM2/CEP12* ratio is crucial to properly determine the amplification status.

**Figure 5:**
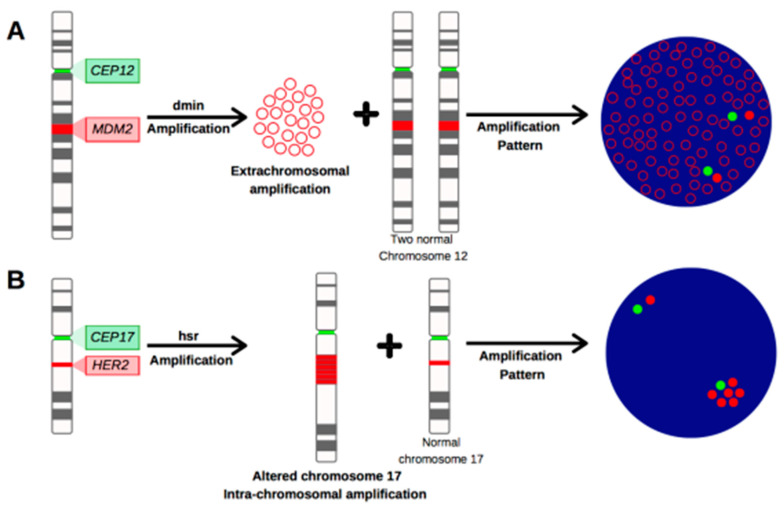
Gene amplification patterns. Amplified DNA can be observed in two different patterns: (**A**) Gene amplification in extra chromosomal entities called double minutes (Dmins). Small fragments of extra chromosomal DNA scattered over the whole nucleus are commonly observed. This pattern is characteristic of the MDM2 gene. (**B**) Gene amplification in intrachromosomal entities called homogeneously staining regions (HSR). HSR are chromosomal segments of various lengths but uniform staining intensity. In the chromosomal region where HSR occur, a segment of the chromosome is amplified or duplicated several times. This pattern is the most common in HER2 amplified cases.

Screening and reviewing the literature regarding *MDM2* amplification, we noticed that several studies used assessment criteria similar to the ones reported in breast cancer *HER2* evaluation guidelines. Overall, we discourage this practice, as these guidelines were tailored for a different setting (breast cancer) and a diverse gene with its peculiar amplification pattern. As we already discussed, *MDM2* amplification is developed through the Dmin mechanism and is usually characterized by the presence of several gene signals (*MDM2* > 10) clustered or scattered over the whole nucleus, with a low centromere number (Figure 5A). Differently, *HER2* amplification mainly occurs with two distinct amplification mechanisms: 30% of HER2-positive breast tumors present a Dmin amplification mechanism with a pattern similar to the ones described for *MDM2* (Figure 5A), but the majority of HER2-positive breast cases (~60%) involve intrachromosomal regions called homogeneously staining regions (HSR) (Figure 5B) [67,68]. HSR have also been identified in breast cancer cell lines, including BT474, SKBR3, and JIMT-1 [69]. HSR are unusual patterns associated with the gain (CEP > 2), loss (CEP < 2), or coamplification of the centromeric region [70]. The differences could depend on the HSR extension and the presence of other cytogenetic aberrations (translocations, inversions, and deletions) involving the gene loci and the CEP. Furthermore, breast cancer *HER2* assessment guidelines are based on different aims which mainly include the prognostic and predictive assessment (the selection of eligible patients for Trastuzumab treatment). Differently, LPS *MDM2* amplification harbors a diagnostic value only, to date. Based on this evidence and distinct characteristics, the application of *HER2* criteria to define *MDM2* status could increase, rather than reduce, inappropriate interpretations and diagnosis.

Our study presents some limitations, mainly represented by the monocentric collection and analysis of our series and the absence of external validation, which is required to independently confirm our diagnostic recommendations.

This effort is especially important if we consider the evaluation of *MDM2* amplification by FISH as a cornerstone of the LPS diagnostic workup, but it is still affected by the lack of consensus guidelines, potentially resulting in different and even misleading approaches. Our study represents the first attempt to solve this controversial scenario and develop formal guidance for the interpretation of this assay to optimize the diagnostic and clinical management of patients with suspected LPS.

## 4. Materials and Methods

This study focuses on the FISH assessment of *MDM2* status in LPS by combining data acquired at our Institution and the evidence published in the literature so far.

### 4.1. Case Series Construction and FISH Analysis

**Figure 6:**
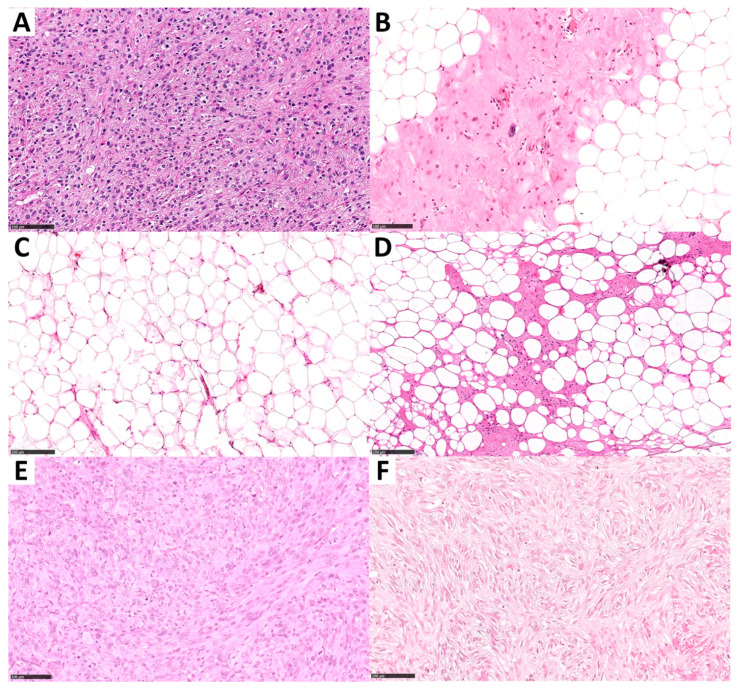
Matched histopathological features of the representative samples presented in Figure 2. Hematoxylin and eosin stain were used to evaluate the histological characteristics of the analyzed tumors and select representative areas for FISH analysis. (**A**) An *MDM2*-amplified DDLPS showing only focal lipomatous areas (the corresponding *MDM2* amplification pattern is shown in Figure 2A). (**B**) An *MDM2*-amplified WDLPS (the *MDM2* amplification pattern of this cases is represented in Figure 2B); (**C**,**D**) Representative images of two lipomas that were analyzed for *MDM2* amplification (the corresponding negative *MDM2* FISH patterns are represented in Figure 2C,D); (**E**) A non-*MDM2*-amplified undifferentiated pleomorphic sarcoma with giant cells (the corresponding *MDM2* and *EGFR* FISH patterns are represented in Figure 2E (including the top inset) and Figure 2F, respectively); (**F**) An *MDM2*-amplified DDLPS with scattered giant cells (the corresponding *MDM2* FISH pattern is provided in Figure 2E (bottom inset)).

Formalin-fixed paraffin-embedded (FFPE) tissue blocks of 83 adipocytic tumors (diagnosed from 2014 to 2019) were retrieved from the archive of the Pathology Unit of the Città della Salute e della Scienza Hospital (Turin, Italy). From each FFPE block, two 4 μm thick tissue serial sections were cut for a tissue adequacy evaluation and FISH analysis. The slides for the tissue adequacy assessment were stained with hematoxylin and eosin and reviewed by two pathologists with soft-tissue expertise (Figure 6).

The slides for FISH analysis were baked overnight at 58 °C and then deparaffinized. Later, samples were treated with the Invitrogen Spot-light tissue pretreatment kit (Invitrogen Corporation, Camarillo, CA, USA) at 98 °C for 15 min, and enzymatic digestion with a protease solution (pepsin) at 37 °C for 45 to 60 min was then performed. Finally, the sections were dehydrated in ethanol of different concentrations for the subsequent hybridization. The hybridization was performed indifferently using two commercially available dual-color probes: MDM2 (green spectrum)/CEP12 (orange spectrum) (Abbott Molecular, Chicago, IL, USA) and ZytoLight SPEC MDM2 (green spectrum)/CEN 12 (orange spectrum) (Zytovision. GmbH, Bremerhaven, Germany) (for consistency, the control probe has always been identified as CEP12). The slides were codenatured in an HYBrite System at 72 °C for 5 min (Abbott) or 75 °C for 10 min (Zytovision) and hybridized overnight at 37 °C. The slides were then washed in a 0.7xSSC/0.3% NP-40 solution at 73.5 °C for 3 min (Abbott) or in a 2xSSC/0.3% NP-40 solution at 73.5 °C for 3 min (Zytovision); then, they were dehydrated in ethanol of different concentrations, air-dried, and counterstained with 6-diamidino-2-phenylindole (DAPI). The presence of polyploidy was identified using a dual-color probe for EGFR (7p11) (orange spectrum; Abbott Molecular) and the centromere of chromosome 7 (D7Z1) (green spectrum; Abbott Molecular).

On each slide, five to ten tumor areas of interest were identified, selected, and automatically acquired with the motorized Metafer 4 Scanning System (Carl Zeiss MetaSystems GmbH. Jena, Germany) equipped with AxioImager epifluorescence microscope (one focus plane for DAPI and nine focus planes for green and red spots). An analysis of the *MDM2/CEP12* and *EGFR/CEP7* probe patterns was performed both with the Metafer 4 software and by counting the *MDM2* and *CEP12* spots on images taken through Metafer 4 and transferred into the Integrated Set of Information Systems (ISIS) software.

Successively, the *MDM2* gene was evaluated on 20 to 200 nuclei in the selected representative areas. Only nuclei with both the *MDM2* and *CEP12* signals were assessed.

An *MDM2/CEP12* ratio higher than two (*MDM2/CEP12* > 2) was considered positive for *MDM2* amplification, as assumed from the available literature [9,18,19,20,21,22,23,24]. Cases with a ratio equal or smaller than two were considered not *MDM2* amplified regardless of the absolute number of copies (e.g., polysomic cases with a relatively high number of *MDM2* copies, but with a high number of *CEP12* copies too).

### 4.2. Literature Analysis

An extensive literature review of the published evidence regarding *MDM2* status in LPS was performed by querying PubMed, Scopus, Embase, and Web of Science databases. Up to 90 papers were identified using the following keywords: (“MDM2”) AND (“amplification”) AND (“FISH”) AND (“LPS” OR “Liposarcoma”). Abstracts of conference presentations, case reports, and non-English written papers were excluded. The title and abstract of the selected papers were then screened and assessed for appropriateness, whereas references were double checked to identify potentially neglected relevant articles and ensure literature research adequacy. From each study, details about study design, material and methods, and FISH analysis outcomes were then evaluated and recorded.

## 5. Conclusions

In conclusion, clinical data and morphologic features are of crucial relevance to approach LPS diagnosis, but molecular techniques, such as FISH cytogenetic analysis, are increasingly required to achieve a conclusive diagnosis, particularly for specific variants such as ALT/WDLPS and DDPLS. Considering its crucial diagnostic role in the LPS diagnostic workup, *MDM2* amplification assessment requires a clearly defined workflow and interpretation criteria.

Based on our experience as a tertiary referral center for LPS diagnostic assessment and considering the current literature evidence, we here proposed a set of criteria for *MDM2* FISH assessment as a step towards the development of consensus-based formal guidelines.

## Figures and Tables

**Table 1:** LPS variants/other lipomatous tumors and corresponding *MDM2* amplification rate.

Case Series	*MDM2* Amplification		
	Amplified	Not Amplified	Ratio (%)
DDLPS	25	2	25/27 (93)
ALT/WDLPS	18	1	18/19 (95)
PLPS	0	3	0/3 (0)
MLPS	0	2	0/2 (0)
SCL/PL	0	5	0/5 (0)
ASCLT	0	2	0/2 (0)
Lipoma	0	25	0/25 (0)
Total	43	40	43/83 (52)

DDLPS: Dedifferentiated LPS; LPS: liposarcomas; ALT/WDLPS: Atypical Lipomatous Tumors/Well-differentiated LPS; PLPS: Pleomorphic LPS; MLPS: Myxoid LPS; SCL/PL: Spindle Cell Lipoma/Pleomorphic Lipoma; ASCLT: Atypical Spindle Cell Lipomatous Tumor.

**Table 2:** Selection of the most relevant published studies evaluating FISH *MDM2* amplification. IHP: in-house probe; SP: single LSI MDM2 probe; DCP: dual color probe.

Sample Size	FISH Probes	N° Nuclei Evaluated	Amplification Diagnostic Cut Off	Year of Publication	Reference
38	DCP	40	*MDM2/CEP12* ≥ 2	2022	[38]
439	DCP	200	*MDM2/CEP12* ≥ 2	2022	[31]
20	DCP	20	*MDM2/CEP12* ≥ 2	2022	[32]
55	DCP	n.a.	n.a.	2021	[39]
35	DCP	n.a.	n.a.	2021	[33]
113	DCP	n.a.	n.a.	2019	[40]
17	DCP	100	*MDM2/CEP12* > 2	2019	[41]
180	DCP	n.a.	*MDM2/CEP12* ≥ 2	2018	[42]
66	DCP	100	*MDM2/CEP12* ≥ 2	2018	[43]
25	DCP	n.a.	*MDM2/CEP12* > 2	2018	[44]
232	DCP	200	n.a.	2017	[45]
101	DCP	40	*MDM2/CEP12* > 2	2017	[46]
18	DCP	n.a.	*MDM2/CEP12* > 2	2017	[47]
140	IHP-DCP	200	*MDM2/CEP12* ≥ 2	2016	[48]
102	DCP	40	*MDM2/CEP12* ≥ 2	2016	[49]
5	DCP	100	*MDM2/CEP12* > 2.0	2016	[50]
5	DCP	n.a.	*MDM2* ≥ 3 *CEP12* = 2	2016	[51]
347	DCP	n.a.	2–4 *CEP12* signals with ≥6 extra *MDM2* signals.	2015	[30]
10	DCP	100-200	*MDM2* ≥ 10 Polysomy *CEP12*: *MDM2/CEP12* ≤ 2	2015	[52]
77	DCP	60	*MDM2/CEP12* > 2	2015	[53]
50	SP	40	*MDM2* > 5.0	2015	[54]
347	DCP	n.a.	*MDM2* ≥ 6 *CEP12* = 2–4	2015	[30]
301	DCP	200	At least 15% of nuclei presenting at least 15 *MDM2* signals per cell	2015	[55]
46	DCP	60	*MDM2/CEP12* ≥ 2	2014	[56]
64	DCP	100	*MDM2* ≥ 5 *CEP12* = 1–2	2014	[57]
172	IHP-DCP	n.a.	*MDM2/CEP12* > 2	2013	[10]
38	SP	n.a.	n.a.	2013	[1]
82	DCP	100	*MDM2/CEP12* ≥ 2.2	2012	[58]
428	DCP	50	*MDM2/CEP12* > 2.0	2012	[59]
12	IHP-DCP	n.a.	n.a.	2010	[60]
54	DCP	n.a.	*MDM2/CEP12* ≥ 2.0	2010	[61]
41	IHP-DCP	40	*MDM2/CEP12* ≥ 2.0	2009	[9]
130	IHP-DCP	40	*MDM2/CEP12* ≥ 2.0	2008	[8]
200	SP	100	*MDM2* > 5 signals/cell	2007	[11]
71	IHP-DCP	100	*MDM2/CEP12* > 3	2006	[62]
21	SP	n.a.	*MDM2* > 2 signals/cell	2000	[63]

## Data Availability

The data supporting the findings of this study are not publicly available due to privacy or ethical restrictions but can be obtained upon reasonable request from the corresponding author.
